# Integrated Care for People with Dementia—Results of a Social-Scientific Evaluation of an Established Dementia Care Model

**DOI:** 10.3390/geriatrics2010001

**Published:** 2016-12-27

**Authors:** Stefanie Richter

**Affiliations:** Department II: Social Infrastructure and Health, Wilhelm Löhe University of Applied Sciences (WLH), Merkurstrasse 41, D-90763 Fürth, Bavaria, Germany; Stefanie.richter@wlh-fuerth.de; Tel.: +49-911-766-069-52; Fax: +49-911-766-069-29

**Keywords:** dementia, integrated dementia care, person-centred care, best practice, qualification profile

## Abstract

Currently, approximately 46.8 million people worldwide and 1.47 million German people are affected by dementia. The rising numbers of cases of people with dementia, the need for complex care and the insufficient care available call for innovative and sustainable solutions both in Germany and many other countries. This article presents results of the social-scientific evaluation of an established care model for people with dementia developed by the professionals as a result of acute problems in care in north-east Germany. In addition to the central elements of the model, the conditions of intersectoral and interprofessional cooperation as well as the qualification profile requirements of the professional groups involved are presented in detail. The results can give suggestions for the organization of integrated care for people with dementia in other countries. Further, the author would hereby like to highlight the gain from the scientific examination of solutions to problems in the field.

## 1. Introduction

A total of 46.8 million people worldwide were living with dementia in 2015 [1]. Currently, approximately 1.47 million people are affected by dementia in Germany. If there is no causal therapy or prevention, up to 131.5 million people with dementia worldwide and 3.02 million cases in Germany are expected for the year 2050 [1,2]. Alzheimer’s disease and other causes of dementia are already amongst the six most common causes of death in Germany [3] (p. 4). They are cost-intensive diseases worldwide [1] and in Germany [4] (p. 13). Although there is no causal treatment at present, sound knowledge about effective strategies to slow down the progressive disease process, relieve symptoms and maintain quality of life does exist. These include

- early diagnosis by the general practitioner and differential diagnosis by the specialist;
- information, education, counselling and advance care planning;
- a combination of medical, pharmacological and non-medical measures tailored to the client and his/her needs that are preventive and resource-oriented, geared towards principles of person-centred care (by Kitwood) and integrate early palliative aspects;
- a structure that integrates outpatient, semi-residential and inpatient care and is managed in the care (case management);
- an environment design adapted to the target group as well as the systemic involvement and support of family caregivers [5,6,7,8,9,10,11].

Key components are already included in German treatment guidelines, such as the S3 guidelines with evidence-based information about prevention, diagnostic and pharmacological and non-pharmacological interventions [5]. However, the reality of the care situation does not yet meet these requirements. Studies point to insufficient or lack of care, regional disparities as well as a lack of integration e.g., [9,12,13,14,15,16,17,18]. These shortcomings can be partly attributed to structural problems in the German health system [19,20,21,22], which complicate intersectoral and interprofessional cooperation, early support in everyday life, and continuity in the care process. In Germany, there is a need for practice solutions which can ensure comprehensive and patient-centred care and can be sustainably implemented. Obviously, other countries are facing similar challenges. Many countries have agreed national dementia strategies. The aim is, among others, to develop appropriate and effective care concepts [8,23].

In recent years, programmes for the research and development of care for people with dementia have been initiated in Germany, in which approaches to solutions have been analysed and developed together with practitioners, e.g., a future dementia workshop of the Federal Ministry of Health or dementia-friendly communities of the Robert Bosch Foundation. The author takes the view that a productive approach is to, on the one hand, systematically analyse, evaluate and condense the overarching findings already established in practice, such as local care network works, and, on the other hand, to develop sustainable solutions in a joint process between the scientific community, professionals, the policy sphere and target groups. Health service research and transdisciplinary research could make an important contribution to improve the care practice for people with dementia.

This article presents results of a qualitative evaluation of an established care model for people with dementia developed by the professionals as a result of acute problems in care in north-east Germany. In addition to the central elements of the model, the conditions of intersectoral and interprofessional cooperation as well as the qualification profile requirements of the professional groups involved are presented in detail. Although the presented model is adjusted to the framework conditions of the German healthcare system, the explanations can give suggestions for the organization of integrated and specialised care for people with dementia in other countries and health care systems. At the same time, the author would hereby like to highlight the gain from the scientific examination of solutions to problems in the field.

## 2. Objective and Methodical Approach of the Study

The aims and the approach of the study are presented below.

### 2.1. Objective

The social-scientific evaluation was aimed at

(a) analysing and describing structure (e.g., areas, points of intersection, and transitions), processes (e.g., care processes, communication and coordination) and the general conditions of the established care model;
(b) working out gaps in care and potential for development with reference to current scientific expertise and the perspectives of the target group;
(c) reflecting on transferability to other regions.

The reasons for this scientific analysis were as follows: The professionals of the care model were interested in systematically describing the successively established structures and optimizing them. The author was interested in investigating a solution in the field, in reflecting methods to analyse practice solutions and to generate knowledge to improve care in collaboration with the professionals and to generate transferable knowledge for the practice and scientific community. The author conducted the study as an independent scientist from April 2012 to March 2014. In the centre of this article are results of the main aim (a). 

### 2.2. Methodical Approach

The scientific challenge consisted of the understanding and the description of the structure, the occurring processes and the framework conditions of a gradually developed care model. The knowledge of the participants fixed and gained during the formation process and the commonplace care activity was not obvious but has to be reconstructed. As for a model from the field, there existed no systematic knowledge, neither about the individual sections and their working methods, the intersectoral and interprofessional coordinated care processes nor about the relevant framework conditions. So, the author decided to conduct a social-scientific-qualitative evaluation to gain generalizable insights, in addition to the description, the assessment and the improvement of the practical experience [24]. An interpretive design in terms of the research strategy of the Grounded Theory [25] was selected. A process according to the Grounded Theory allows systematic knowledge and theory generation based on empiric data. It is applied to fields of research where no assured knowledge is available. A circular research process directed by theoretical sampling and abductive research logic is typical [26]. The selection of the data collection methods is aligned with the subject. Different methods can be combined in the research process. For the data analysis, Strauss and Glaser propose the methods of coding and permanent comparison [25], however, further interpretive procedures are also possible [27].

### 2.3. The Implementation

Data collection, data analysis and knowledge generation were carried out according to the theoretical sampling. Thus, an initial data collection was carried out, the data were analysed and on this basis, new criteria were determined for the following collection.

For the *data collection* different methods were used:

(a) Open, guideline-based interviews were conducted with the professionals of the care model. Among others, the experiences during the common development process of the setting, the main duties and the underlying concepts, care processes, interfaces and coordination processes, conditions for a successful cooperation and financing frameworks were inquired. Heads of each care section were available for repeated interviews.
(b) Narrative-generating interviews were conducted with three family caregivers. The aim was to reconstruct the illness trajectory and the care context of the family member with dementia from the relative’s point of view. They had shown interest and agreement.
(c) The participation of observations in the different care sections and in comprehensive team meetings took place to understand the procedures, the implementation of principles, workflows, interface management and communication between the professionals.
(d) Documents such as self-descriptions, working aids or quality standards were checked and analysed.
(e) During the research process, it was necessary in some cases to discuss some aspects in group discussions, e.g., the division of work in assisted living. Furthermore, based on case studies, professionals were asked to explain in writing or orally, their tasks, procedures and interactions in the care process for their clients.

The first step was the first survey phase. Here, heads of each care section were interviewed based on guidelines and the participation of supervisions of comprehensive team meetings took place. The perspectives of all partners should be included: medicine, psychology, nursing, support, rehabilitation, day care, assisted living, service, administration and management. The interviews were recorded and subsequently either taped and transcribed, or recorded in writing. The observations were recorded in writing. After the first survey phase, the data were evaluated and new questions for a further inquiry and observation round were generated.

The *data analysis* was performed using qualitative content-analysis [28] and code technique [25]. On the one hand, the statements were analysed, classified and compressed based on criteria, on the other hand concepts were identified, abstracted and described in open coding mode. It was characteristic that during the process of theoretical sampling, the data collection has always been adjusted to the subject; that the findings from the interviews, the observations and document analyses related to one another (triangulation); and that the sampling has been stopped at the point where there were no more questions (theoretical saturation). There were five survey phases during several days, in total. 

In parallel to the fieldwork, a *literature review* was compiled consolidating the international expertise on the care of people with dementia. Particular attention was paid to the standards of diagnosis, counselling, therapy, family support and case management. The overview was used to reflect on the general status quo of care and to submit development proposals to the professionals.

The results were *transferred* in the form of feedback sessions and group discussions with the professionals. The outcomes of the discussions were mutual knowledge, the validation of results, reflection on professional action and strategy development. 

The research process was reflected by a *memo in writing* [25]. Decision making processes, e.g., field access, method selection, sampling were documented in the memo; ideas and categories were recorded and described; problems in the research process and the own role were reflected. 

The results of the research process are consolidated pictures, case studies and comprehensive findings see [11]. The following presentation of selected results is based on these descriptions. The objective is to develop comprehensive findings and to give suggestions for the organization of care practice.

## 3. Results

### 3.1. Initial Situation—Pressure Caused by the Problem, Opportunity for development and Positive Network Experiences

The starting point is a medium-sized town in north-east Mecklenburg-Western Pomerania (East German federal state), in which the demographic change was visible at an early stage, made worse by the departure of younger sections of the population since the collapse of the system in 1989. In 2013, already 25% of the town’s 57,301 residents were aged 65 or older [29]. The nationwide average in Germany was 21% in 2013 [30] (p. 17).

The momentum to develop specialised care for people with dementia emerged in the course of the rising number of cases and the limited possibilities of action in an outpatient social-psychiatric specialist practice: the number of older clients with cognitive disabilities was increasing steadily. However, the psychiatrist was only brought in at very late stages, which suggests insufficient screening on the part of the referring general practitioners and access problems on the part of the clients; the practice was not in line with the target group from a space, diagnostics or staff perspective; cooperation with external services often broke down in the communication, or specialist care discontinued after a transition to a hospital or care home. Given the right to care that is consistent with guidelines and taking into account the population-related need, the doctor saw the solution in the creation of a memory clinic with a comprehensive care network. The psychiatrist was able to draw on these experiences and the partners in a psychosocial health network, which encouraged the concept and implementation. The specialised care model was founded in 2009 and developed gradually. The psychiatrist has specialised in geriatric psychiatry since 2009.

### 3.2. The Care Model with the Central Components

The dementia care centre is geared towards people with memory disorders and their family caregivers. The objectives are an early diagnosis as well as complete and coordinated care designed for the needs and requirements of the clients and relatives throughout the entire course of the disease. To do this, the geriatric psychiatrist works closely with professionals in support, nursing, rehabilitation, day care and assisted living under one roof (Subsequently, the professionals of the dementia care centre are described as partners in terms of cooperation partners.), as well as with other local professionals. In contrast to the majority of memory clinics established at clinics, which mainly focus on diagnosis and drug therapy [31], the diagnostic and medical services are extended through a wide range of non-medical services and coordinated on a case by case basis.

**Figure 1:**
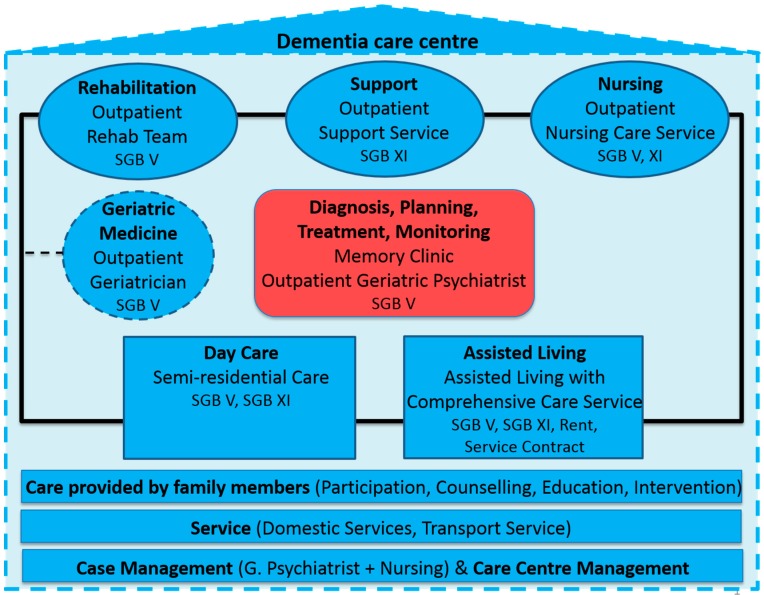
The central elements of the dementia care centre and the financial base in Germany (Book of social insurance). (Modified and adapted from ([11], p. 88)).

The central elements of the care model are shown in Figure 1 and are outlined below (for more detail, see [11]). 

*Memory clinic*: The client’s access is via the geriatric psychiatry consultant practice. This is where differential diagnosis, treatment planning, counselling, medical treatment, semi-annual monitoring and care coordination take place with partners. The geriatric psychiatrist accompanies the clients from the diagnosis throughout the entire course of the disease as an outpatient, in day care, as well as in the assisted living accommodation, and assumes the management tasks. Initial contact as well as all subsequent meetings generally involve the family carers. This especially serves the health care of the relatives in addition to counselling and the sustainable implementation of the treatment plan. In this way, the doctor can counteract the first signs of overload at an early stage through targeted strategies. The geriatric psychiatrist works systematically on the points of intersection with partners in the care environment and with other local service providers. For example, the geriatric psychiatrist informs the general practitioner about the diagnosis, the treatment plan as well as any later modifications, and coordinates the drug therapy with him. If the client is in day care, the follow-up appointment with the doctor involves the day care manager (a specialised nurse). The joint discussion between the client, family members, the geriatric psychiatrist and the day care manager provides a comprehensive picture of the status quo and ensures that everyone is equally informed and that changes in the care plan, e.g., fall prevention in day care, are implemented immediately. In assisted living, weekly multi-disciplinary case discussions take place with the geriatric psychiatrist so that medical matters can be cleared up immediately and medical therapeutic measures can be initiated, implemented and monitored in terms of their effectiveness. The digital client file also facilitates information and coordination with the professionals within the care centre. The systematic division of care tasks with the nursing staff relieves the pressure on the doctor. The nursing staff of his/her practice team take care of, e.g., test diagnosis, education, counselling and coordination tasks, such as recall and coordinating with the general practitioner and partners involved. In day care, the day care manager (a specialised nurse) takes care of the smooth implementation of the care plan or managing the transition of a day care client, e.g., into a hospital. The same applies for nursing care in assisted living.

*Rehabilitation*: Occupational therapists (An occupational therapist is targeted to the therapeutic support of people who are restricted or threatened in their capacity to act. The support of motor, psychological and cognitive-functional capacities and the environmental adaptation and consulting are targeted to the maintenance of the capacity to act, participation and quality of life.), physical therapists and athletic trainers of a local rehabilitation centre work as a permanent rehabilitation team in the care centre so that effective rehabilitation measures can be implemented from the outset. Rehabilitation is a fundamental part of the overall concept, both in the form of group and also individual services. Each day, the care client and assisted living resident receives an individual rehabilitative therapeutic programme geared towards his/her needs and participates in group activities. In the advanced stage of the disease, the occupational therapists provide palliative care to the resident. In the care centre, the client can be accompanied throughout several stages of care by the same therapists from the beginning until they die. This avoids interruptions in the care and promotes relationship building as well as a lengthy continuity of skills. Working in a team with other professionals provides therapists with, e.g., case-related coordination and mutual support and relief.

*Low-threshold support* (Support services are low-threshold offers of support to cope with everyday life, such as daily planning, household tasks and to relieve family members from the pressure of supervision. They can be applied for when the first permanent limitations on handling daily life occur (Book XI of the German Social Security Code).): An outpatient support service provides low-threshold support services. Employees of a local nursing service have specialised as a solid team for these tasks. Systematic support is an important resource for the early encouragement and support in home life as well as the relief of pressure of family members. The support services are structured accordingly (assessment, goal-setting, planning, implementation and evaluation) and the professionals work looking ahead. If the support staff identifies, e.g., risks from inadequate housing design, then it advises the relatives or informs the geriatric psychiatrist. In assisted living, support workers are hired on service contracts in order to support the structuring and shaping of everyday life. They work together as a team with the outpatient nurses and rehabilitation therapists and provide support for family members.

*Nursing care* (Long-term outpatient nursing services include basic nursing care under Book XI of the German Social Security Code (e.g., assistance with personal hygiene and nutrition), counselling and domestic help (cooking, shopping and cleaning). Utilisation implies someone is in need of long-term care. A person in need of long-term care is, according to Book XI of the German Social Security Code, someone who is restricted on a continuing basis due to an illness or disability in performing ordinary recurring activities in the course of everyday life. In addition, there is nursing treatment according to Book V of the German Social Security Code. It requires a doctor's prescription and is usually limited to four weeks (e.g., wound care, medication).): An outpatient nursing service undertakes the preventive, rehabilitative, basic treatment, and palliative care at home, as well as in assisted living. The nursing staff are mostly involved and specialised in the care of people with dementia. The same applies for the rehab team, i.e., that the nursing service can tend to the client from the first care stage to the end of life despite a transition into assisted living accommodation, and can ensure continuity. Primary nursing and client-centring is implemented both in home life and in assisted living according to Kitwood. Although a change in the primary nursing staff does take place during the transition to assisted living, information is systematically redirected, supported by coordinated digital files and assessment tools. This increases the opportunity for suitable subsequent nursing and reduces transitional crises.

*Day care* (In Germany, there is the possibility of semi-residential care in the form of day care. This is where clients can be professionally cared for and looked after during the day.): Day care with a solid team of nursing, support and rehabilitation specialists provides clients with the structuring of daily life on week days, individual and group therapy, support, social integration and medical care through appointments in the memory clinic. The day care manager undertakes, in close coordination with the geriatric psychiatrist, case-related coordination and the coordination of care with the professionals involved, the general practitioner and family members. For cases involving a move into assisted living or hospital, it manages the transition. The concept focuses on people with dementia and involves family members through active participation, information, self-help and exchange of experiences. A transport service ensures access for clients who are no longer mobile.

*Assisted living*: A living arrangement with dementia-friendly designed flats and communal areas. The goal is to maintain independence, self-determination and social participation for as long as possible and to avoid a transition into a nursing home. The fundamental elements are (a) the spatial design and concept geared towards dementia; (b) the combination of support, medical, therapeutic and nursing services; and (c) the role of those with dementia and their family members as tenants (possibility of co-decision and participation). The advantage is that the client can be assisted more during a transition to assisted living by the existing outpatient services and that the range of services can be gradually extended according to the client’s needs. A care plan is created for each resident, which is reviewed and adjusted in the weekly multi-disciplinary case meetings of the team of the geriatric psychiatrist, nursing and support staff and rehab therapist. The resident has a team for nursing, support and treatment. Family members can get involved if they wish to do so to help shape everyday life and they receive offers of advice, information and exchange. The internal case management relieves the pressure on relatives during the transition into assisted living and afterwards. The cooperation with a specialised outpatient palliative care team and a hospice association ensures appropriate support at the end of life in assisted living.

*Geriatric medicine*: A local geriatrician practices in the care centre on fixed weekdays. There are multiple benefits: the general medical care opens up the establishment for the older residents of the district and thus reduces the fear of entering a place; early detection and early access to diagnosis, counselling, treatment and care are promoted; short distances enable direct dialogue, particularly on geriatric issues; clients of the care centre have the opportunity of elderly medical care on-site, especially those whose general practitioner care is no longer possible in assisted living.

*Care provided by family members*: The inclusion of family members or caregivers is an integral part of the overall concept. It begins with the first appointment in the memory clinic and focuses on participation, information, counselling, competence, relief and (crises) intervention. For example, modularized training is carried out for family members, family meetings and joint activities are held quarterly, and collaboration is established with a gerontopsychiatric day clinic. Here, caregivers in crisis situations can be strengthened and relieved by simultaneous day care of the sick family member.

*Local collaborative network:* The professionals of the dementia care centre may not provide comprehensive care in every individual case. The client and family members have the freedom of choice and may opt for other local service providers, such as a nursing service. With the goal of achieving seamless and case-related care, the care centre belongs to a collaborative network of other local partners and institutions. The systematic development of cooperation structures is a basic principle of the dementia care centre partners. For example, general practitioners play a central role in successful care: they refer clients, and measures must be coordinated with them as the care progresses. In the overall concept of the integrated care model, cooperation with the general practitioner is systematically promoted, e.g., through information, structured patient letters and the involvement in decisions, or by coordinating appointments and providing diagnostic rooms in assisted living. After the first few years it can be observed that general practitioners now refer their clients at the first sign of cognitive impairments and are interested in a division of labour. The geriatric psychiatrist observes these signs by values gained through diagnostic tests on new patients and by the quality of the direct collaboration with the general practitioner.

*Special building structure:* The geriatric psychiatrist, the support and nursing staff, the rehabilitation team, and day care and assisted living are grouped in a special building structure. The memory clinic follows on from the entrance and is located directly adjacent to the assisted living and day care facilities. The construction, design and conception of the entire centre are especially tailored to dementia care. The building is visible in the residential district and can be reached by car and bus. The obvious appearance of the building as a dementia centre sensitizes the inhabitants of the municipality. 

*Care financing in the German Health System:* In Germany, more than 90% of the population is covered by social insurance. They are entitled to benefits according to Books V (Statutory health insurance) and XI (Statutory long-term care insurance) of the German Social Security Code. For basic information about the German health system, see [32]. In principle, the medical and non-medical services of the presented integrated care model are provided in the framework of the social security system (Books V and XI). Therefore, any client with social security can make use of them. They have to co-pay a daily rate in day care or the rent in assisted living. However, both can be supported, if necessary, via social welfare (Book XII of the German Social Security Code).

### 3.3. Conditions for Collaboration and Quality of Care

Some aspects are mentioned below that could be presented in detail as being significant for interprofessional and intersectoral cooperation, as well as for the design of care tailored to the individual case in the course of the disease.

- *Being all grouped into one place* allows clients and family members to access a broader scope of information, counselling and help and facilitates the partners on-site to communicate, coordinate and connect. In assisted living, the time taken up for arrival and departure is reduced for out-patient services (e.g., nursing) when providing care to several residents. They use the time saved to work with the client.
- *The dementia-friendly building and the design of the environment* promote the independence and orientation of clients and support the work of the professionals. For example, in assisted living, the flats and common areas are connected by a circuit and the garden is accessible in the middle (atrium) and can be seen from everywhere so that the residents can move independently indoors and outdoors and the professionals also have everything in sight.
- *Collaborative agreements* between the partners as well as the development, implementation and regular evaluation *together with practiced objectives, culture and standards* are the basis of the collaboration and quality of care. The learning processes take place for the partners as early as the negotiation of the common shared objectives, culture and standards, which advance the interrelated actions in the care as well as the development of a collective identity. The agreed basic principles that guide the perception and action apply in everyday care. These include, e.g., the principles of working with the clients and their family caregivers, of cooperating with external partners such as the general practitioner, as well as of teamwork or standards of human resources development, quality assurance and public relations.
- The *definition and transparency of each field of work and expertise of the partners and departments* reduces the risk of duplicate structures and competition and promotes the implementation of teamwork as well as a specific routing for questions from other areas.
- *Information and communication technologies*, such as the digital client file of the geriatric psychiatrist with access rights for those involved in the care process or coordinated assessment and documentation systems, support a smooth information and communication flow as well as knowledge integration.
- The *establishment of comprehensive exchange and development forums* is, in addition to the structures of case-related interprofessional dialogue, important for the joint maintenance of the network, for the development and implementation of strategies and standards or for problem and conflict resolution. This includes, e.g., regular management meetings or quality circles. Experience in transparency, participation, appreciation, negotiating at eye level and achieving overarching goals promote a collective identity. Similarly, the collective design of the centre has proven to contribute to the sense of identity.
- The common *person-centred practice, modularised service structure, case management* and *monitoring* facilitate the provision of care tailored to needs. *Defined care paths and interfaces*, as well as *structured transitions* ensure continuous and coordinated care. The risk of gaps or interruptions in care in the course of the disease is reduced simultaneously through the comprehensive range of care services and the cross-sectoral provision of services, e.g., from home in assisted living. The geriatric psychiatrist, as a gatekeeper and case manager, in tandem with the nursing care, ensures a needs-based, coordinated and continuous provision of care.
- *Care centre management* supports the collaboration of partners and undertakes, e.g., tasks such as controlling, moderation of interprofessional working contexts (e.g., quality circles), public relations or the management of development processes.
- *The opening of the centre* in the community, on the one hand through the geriatric practice, on the other hand through offers, such as exercise groups for elderly people with or without dementia in the district as well as information events, promotes public awareness of the issue and reduces fear of the unknown.

Working with people with dementia and their family members as well as in interprofessional environments requires a wider spectrum of knowledge and skills of all the professional groups involved. Successful team work requires the appropriate attitude (such as openness, appreciation, willingness to learn), interdisciplinary skills (e.g., in communicating, interacting and negotiating), as well as knowledge beyond their own area of expertise about risks, needs and care. The professionals are thus in a position to identify changes in the client early on and to take steps beyond their own competence.

### 3.4. Extended Task Profiles and Skill Requirements of the Doctor and Nursing Care

Extended task profiles, especially for the nursing care and geriatric psychiatrist, become apparent in the implementation of coordinated and comprehensive care in the course of the disease. The acquisition of mere knowledge about dementia has proved insufficient. 

*The nursing role* encompasses, in addition to basic care and treatment, above all, the early detection and prevention of risks and deterioration (e.g., test diagnostics, fall prevention, cognitive training); effective physical, psychological and social activation and empowerment to promote well-being and to maintain competence (e.g., in the area of support, day care); counselling, information and education (e.g., in family work); environment design and purchasing assistance systems; palliative care as well as networking and management (e.g., case management, transition management, management of day care). The implementation requires the appropriate qualifications but also factors such as an expanded concept of the need for care in Germany, nursing concepts specific to dementia, effective nursing measures or an institutional recognition of the work with people with dementia.

*Geriatric Psychiatrist actions* include tasks such as the medical history, differential diagnosis as well as the diagnostic consultation with information, prognosis and forward-looking advice; planning the care in coordination with the client, relatives and the professionals involved; if required, the initiation of non-medical measures by providing information and mediation on site; specialist medical treatment taking into account co-morbidities, multi-morbidity and multi-medication; out-patient or in-patient monitoring with the inclusion of the professionals involved; the transfer of treatment-related information and recommendations to internal and external partners, such as the general practitioner; the systematic documentation and pooling of information in the digital client file; the continuous advice, information, and health care of family members as well as the representation of professional, medical, guideline-oriented expertise in the context of cooperation. In addition to a professional specialisation, when working with clients and cooperating with non-medical professionals, more knowledge and skills become relevant: social and communication skills, e.g., for communicating the diagnosis and consultation; planning, coordination, presentation and negotiation skills, e.g., for the sensitive balancing of own and external views when talking with clients and their families, or in the management between clients, family members and professionals; positive attitude regarding coproduction, i.e., the equal participation of both professional and civil, organised or informal, partners; team orientation; knowledge about non-medical care needs, non-medical services, offers and local structures as well as on the socio-legal framework.

## 4. Discussion and Outlook

Care epidemiological studies point to a need to develop the care of people with dementia in Germany. An analytical look into the practice, however, shows that there are already healthcare approaches which meet the required standards. The presented example of practice provides a comprehensive client-centred care for people with dementia for the entire course of the disease. The involved partners realize standards such as “good care” as they are described in the introduction:

- early access to the memory clinic is encouraged through collaboration with the general practitioners and the local presence of the centre; based on this, the chance for an early and differentiated diagnostic and treatment increases;
- education and counselling, care planning and continuous progress monitoring by the geriatric psychiatrist accompany the medical differential diagnosis;
- medical and non-medical measures are coordinated on a case by case basis and adapted over time;
- the work is aligned with the principles of dementia care and focuses particularly on resource-conserving and preventive strategies, supplemented by palliative care in advanced stages;
- outpatient, semi-residential and residential structures are integrated and transitions are structured both within the care setting as well as at other local facilities;
- both the concepts and the spatial design are tailored to the target group;
- from the outset, family caregivers are involved and relieved.

However, health-economic studies and a study which investigates the outcome based on clinical parameters compared to the standard care would be necessary to make reliable statements regarding effectiveness and efficiency. However, various observations point to a high quality of care: the early access as a result of a higher sensibility of general practitioners and the population. Many outpatient clients continue to live at home for a long time. In assisted living, residents in advanced stages are mobile (instead of confined) without measures restricting their freedom, such as bed rail or fixtures. Case reconstructions based on interviews with family caregivers indicate that early diagnosis, information, continuous care and the accompaniment of family caregivers make things easier and delay or prevent a transition into a care home or assisted living. Crises situations are, however, developments where there is no diagnosis and access to support fails [11] (pp. 16–56).

It is clear that successful interprofessional and intersectoral care requires structures and collective development processes, and brings with it new tasks and requirements for all the partners involved. Although the geriatric psychiatrist takes on the role of gatekeeper, the provision of the service is co-produced and team-oriented. For the doctor, there are multiple benefits despite increased effort. He can initiate interventions in medical care and thus increase the success of treatment, and help ensure effective care in accordance with guidelines in terms of quality of life, preventing care home transitions and unburdening family members until death. Teamwork and case discussions enable him to become more knowledgeable and mean that measures can be coordinated, modified and reviewed. The division of labour (e.g., psychosocial counselling and coordination through nursing), the availability of options for action beyond the medical repertoire and the involvement of a multi-professional team can provide relief. The same is true for other professionals who can handle complex problems of clients better as a result of collaborating and take some pressure off themselves. The cross-sectoral design increases the chance of long-term support of the clients.

Beside a systematic description and elaboration of comprehensive findings (see objective (a)), development concerns have been discussed and initiated with the partners, for example, the establishment of a gerontological psychiatric day-care clinic, offers of self-help for persons with Mild Cognitive Impairment (MCI) or the implementation of a 24-hours-emergency call. For the transferability of the model to other regions, structural and substantial framework conditions have been developed (see [11], pp. 257–261). It would be preferable to verify the effectiveness, the economic efficiency and transferability based on the participation of different disciplines and to evaluate the implementation in a second region. With the selected qualitative design, it was possible to reconstruct structures, procedures and intersectoral and interprofessional coordination processes, to generate comprehensive theoretical findings, to assess together with the professionals the status quo with reference to the current scientific level of knowledge and to work out the development opportunities.

The qualitative analysis of the care model developed gradually in practice for people with dementia shows that a discussion of problem-solving in the field is useful. In the context of the demographic and epidemiological change, professionals are confronted with an increasing discrepancy between the requirements of complex care and their own limited room for manoeuvre. They have to develop innovative solutions in fixed contexts (social, regional, social structural). However, they lack the resources and methods to investigate, describe and disseminate approaches that are often works-in-progress. They remain “undiscovered”. Scientists have the necessary methods. Certainly, open qualitative procedures are an advantage in the initial stage, facilitating understanding and discovery. Through mutual exchanges, the potential for developments can also be worked out together. However, this requires acceptance, appreciation and being open to the language and logic of others.
